# Chitosan-Based Hydrogels for Controlled Delivery of Asiaticoside-Rich *Centella asiatica* Extracts with Wound Healing Potential

**DOI:** 10.3390/ijms242417229

**Published:** 2023-12-07

**Authors:** Katarzyna Witkowska, Magdalena Paczkowska-Walendowska, Tomasz Plech, Daria Szymanowska, Bożena Michniak-Kohn, Judyta Cielecka-Piontek

**Affiliations:** 1 Department of Pharmacognosy and Biomaterials, Poznan University of Medical Sciences, Rokietnicka 3, 60-806 Poznan, Poland; witk.katarzyna@gmail.com (K.W.); mpaczkowska@ump.edu.pl (M.P.-W.); daria.szymanowska@up.poznan.pl (D.S.); 2 Department of Pharmacology, Medical University of Lublin, Radziwillowska 11, 20-080 Lublin, Poland; tomasz.plech@umlub.pl; 3 Department of Pharmaceutics, Ernest Mario School of Pharmacy, Rutgers—The State University of New Jersey, Piscataway, NJ 08854, USA; michniak@pharmacy.rutgers.edu; 4 Center for Dermal Research, Rutgers—The State University of New Jersey, Piscataway, NJ 08854, USA

**Keywords:** *Centella asiatica*, asiaticoside, chitosan, hydrogel, wound healing

## Abstract

*Centella asiatica* extract is a valued plant material with known anti-inflammatory and anti-microbiological properties. Using the Design of Experiment (DoE) approach, it was possible to obtain an optimized water/alcoholic extract from *Centella asiatica*, which allowed the preparation of the final material with biological activity in the wound healing process. Studies on the novel applications of *Centella asiatica* in conjunction with the multifunctional chitosan carrier have been motivated by the plant’s substantial pharmacological activity and the need to develop new and effective methods for the treatment of chronic wounds. The controlled release of asiaticoside was made possible by the use of chitosan as a carrier. Based on the findings of investigations using the PAMPA skin assay, which is a model imitating the permeability of actives through skin, this compound, characterized by sustained release from the chitosan delivery system, was identified as being well able to permeate biological membranes such as skin. Chitosan and the lyophilized extract of *Centella asiatica* worked synergistically to block hyaluronidase, exert efficient microbiological activity and take part in the wound healing process, as proven in an in vitro model. A formulation containing 3% extract with 3% medium-molecular-weight chitosan was indicated as a potentially new treatment with high compliance and effectiveness for patients. Optimization of the chitosan-based hydrogel preparation ensured the required rheological properties necessary for the release of the bioactive from the chitosan delivery system and demonstrated a satisfactory antimicrobial activity.

## 1. Introduction

Currently, it is thought that six million people worldwide experience some form of chronic wound. The primary cause of these wounds is an inflammatory condition, which eventually jeopardizes the tissues’ dermal and epidermal integrity. The most prevalent causes of wound infections resulting in a delay in healing are bacterial strains typically found in the human body, such as *Pseudomonas aeruginosa*, *Escherichia coli*, and *Staphylococcus aureus* [1]. These infections result in prolonged inflammation, which is a major cause of chronic wounds on a cellular level [2]. Therefore, there is a need to search for improved formulations with new raw materials that will produce therapeutic multidirectional effects.

Traditional medicines are potentially a secure and affordable approach for treating burns and wounds; thus, they have drawn the attention of current researchers [3]. One of the most interesting plant materials is *Centella asiatica*. The most important compounds of this plant material include triterpene acids, termed sapogenins (asiatic and madecassicacids), and andtriterpene glycosides (asiaticoside A, asiaticoside B, madecassoside) [4]. There are reports that both *Centella asiatica* extract and asiaticoside show activity in tissue regeneration [5] and the wound repair process by promoting fibroblast proliferation and collagen synthesis [6].

In order to control the delivery of active compounds to the site of action, it is necessary to provide them to the patient in an appropriate pharmaceutical dosage form. In recent years, hydrogels have gained a lot of interest in this field. Most of the requirements for contemporary wound dressings are met by hydrogels, including the capacity to absorb wound exudate, maintenance of a humid environment, thermal insulation, the presence of antibacterial qualities, security, ease of removal from the wound surface, and patient-friendly dressing changes [7]. Networks made of natural (polysaccharides like alginate, cellulose, chitosan, and pectin or proteins such as collagen or gelatin chains) or synthetic (poly(vinyl alcohol), polyacrylamide, poly(ethylene oxide) and poly(ethylene glycol)) polymeric materials are used to form hydrogels [8]. Among these, chitosan (CS) has the most interesting properties. CS is obtained by partial deacetylation of insoluble naturally available chitin. CS has been reported to exhibit biodegradability and biocompatibility [9], and controlled release of active compounds [10].

There are scientific reports about the possibility of forming *Centella asiatica*-rich polyvinyl alcohol/polyethylene glycol (PVA/PEG) [11] or gelatin/chitosan/nonwoven fabric composite hydrogels [12] for accelerating wound healing. Of other modern forms, CS-based microneedles [13] and electrospun gelatin nanofibers [14] have been proposed as well.

The aim of the current research described in this paper was to optimize, for the first time, the ultrasound-assisted extraction (UAE) of *Centella asiatica*. In the second stage of the research, the process of producing CS-based hydrogels was optimized, taking into account the properties of three different molecular weights of CS. This is the first approach using a DoE to optimize the composition of hydrogels containing *Centella asiatica*. Thanks to the proposed support in conducting all research in accordance with the Design of Experiment (DoE) methodology, it was possible to obtain the greatest possible information about the processes with a limited number of actual experiments conducted in the laboratory in in vitro conditions.

## 2. Results and Discussion

Optimizing the extraction process is one of the key elements in the development of natural products. Previous research on *Centella asiatica* extraction involved the use of maceration, distillation, and Soxhlet [15], ultrasound-, and microwave-assisted extraction (MAE) [16,17], as well as subcritical water extraction [18]. Ultrasound-assisted extraction (UAE) is known as a method to recover a high yield of phenolic and flavonoid compounds with the highest antioxidant activity [15]; however, studies using this method [16] did not take into account the assessment of the biological properties of the obtained extracts; therefore, this aspect was also included in the presented study.

**Figure 1:**
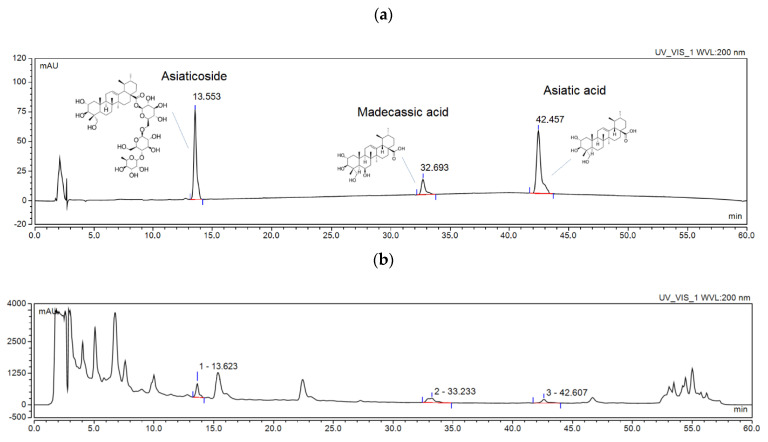
Chromatogram of standards (**a**) and extract E15 (**b**).

The independent variables of UAE were the methanol percentage (50–90%), extraction temperature (30–70 °C), and a time of 30–90 min (three cycles). Based on the experimental matrix, 15 extracts marked as E1–E15 were prepared. Each of these variables affected four dependent parameters: the sum of asiaticoside, madecassic acid and andasiatic acid content measured using the HPLC method (Figure 1; Table S1, Supplementary Material); the total phenolic content (TPC); the antioxidant activity; and the anti-inflammatory activity. The results are collected in Table 1.

Figure 1 shows the chromatograms for standards (AS c = 396.00 µg/mL; AA c = 229.60 µg/mL; MA c = 52.00 µg/mL) (a) and for extract E13 (b).

In the case of the sum of active compounds, the most important is the percentage of methanol used in the extraction mixture (Figure S1, Supplementary Material), while the TPC is statistically significantly influenced by the temperature of the process (Figure S2, Supplementary Material). Interestingly, Thong-on et al. described that for UAE, the ethanol percentage had a negative effect on the contents of asiaticoside and asiatic acids, but had no effect on the madecassic acid content [16], which indicates the importance of the extractant used (methanol or ethanol).

**Figure 2:**
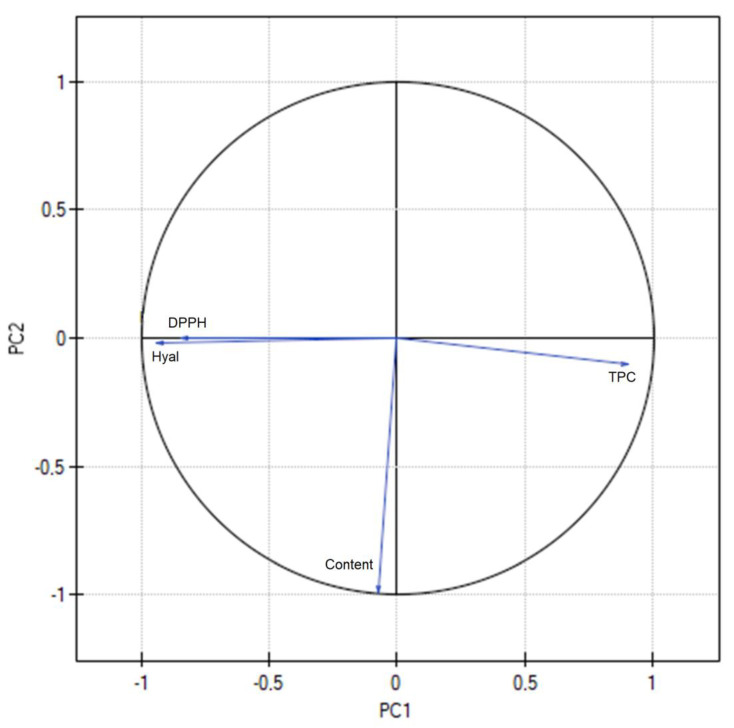
PCA for phytochemical characteristics of extracts.

The antioxidant activity of *Centella asiatica* was evaluated by its ability to scavenge DPPH free radicals. The *C. asiatica* extract showed a statistically significantly higher antioxidant activity when using a higher content of methanol in the extraction mixture (Figure S3, Supplementary Material). Previous research suggests that the presence of compounds with free hydroxyl groups may be the cause of the extracts’ potent antioxidant effects. Given this, flavonoids are significant antioxidant agents because they contain a number of hydroxyl groups that act as hydrogen donors and have a perfect structure for scavenging free radicals [19]. This, in turn, is in strong correlation with the sum of active compounds (AS, AA, MA) and TPC, where the higher activity of extracts is shown by those with a greater number of phenolic compounds (Figure 2). When assessing the antioxidant power of the extract, it can be assessed as an average. Of course, when compared to one of the most active substances, i.e., ascorbic acid (IC_50_ = 8.50 ± 0.59 µg/mL), the activity is much lower. In the case of plant raw materials, an activity below 1 mg/mL is satisfactory and indicates adequate potency.

When analyzing anti-inflammatory activity, it was shown that activity is higher when the temperature is lower (Figure S4, Supplementary Material). The anti-inflammatory properties of *Centella asiatica* extracts are related to saponins, especially asiaticoside’s ability to inhibit of the cyclooxygenase and lipoxygenase activity and inhibit proinflammatory cytokines [20]. So, again, there is strong between of anti-hyaluronidase activity and the sum of active compounds (AS, AA, MA) and TPC, where the higher activity of extracts is shown by those with a greater number of compounds (Figure 2). Obviously, a very strong correlation was observed between antioxidant and anti-inflammatory activity, which shows that both are strongly related to the content of active compounds (Figure 2; Table S2, Supplementary material).

On the basis of the experimental studies and statistical analyses, it was possible to predict a model and indicate the optimal parameters of the extraction process. The optimal UAE parameters for *C. asiatica* extraction were 70% methanol as the solvent at a temperature of 70 °C for 3 cycles for 60 min (Figure S5, Supplementary Material). Importantly, in no case was the extraction time a statistically significant factor; therefore, an intermediate value was selected.

The extract was then prepared under the optimized conditions listed above and subjected to a lyophilization process to obtain a dry extract, which was used for further research. The optimized freeze-dried extract was standardized using a previously developed HPLC method and the contents of AS, AA and MA were obtained as 390.96 ± 2.24 µg/100 mg, 18.50 ± 0.08 µg/100 mg, and 3.89 ± 0.02 µg/100 mg lyophilized extract, respectively (sum of AS, AA and MA: 413.35 ± 2.34 µg/100 mg lyophilized extract), with TPC = 206.86 ± 8.56 mg GAE/1 g plant material. In the case of the antioxidant activity assessed using DPPH radicals, an IC_50_ = 144.82 ± 5.20 µg/mL was obtained, and in the case of anti-the inflammatory activity expressed as the ability to inhibit the hyaluronidase enzyme, IC_50_ = 67.78 ± 4.92 mg/mL.

The activity of the optimal extract was evaluated using the procedures described in the literature to assess the microbiological activity of *C. asiatica* extracts, which gave the reference value for the subsequent part of the research (Table 2).

According to a number of literature reports, CS is a carrier with strong adhesive capabilities. It was established that combinations of plant extracts or polyphenolic chemicals have a synergistic impact when combined with chitosan, such as *Calendulae flos* extract [21]. Chitosan delivery devices were obtained in order to ensure prolonged contact of the *C. asiatica* lyophilized extract with the wound and ensure the possibility of creating synergistic biological activity. A Box–Behnken statistical screening design was used to statistically optimize the formulation factors and evaluate the main effects. The independent variables of hydrogel preparation were the CS molecular weight (100–1000 cps), CS concentration (1–3%) and extract concentration (1–3%). On the basis of the experimental matrix, 15 hydrogels marked as H1–H15 were prepared. Each of these variables affected six dependent parameters: asiaticoside release in µg/cm^2^ and %, asiaticoside penetration through skin, hydrogel viscosity, and antioxidant and anti-inflammatory activities.

**Figure 3:**
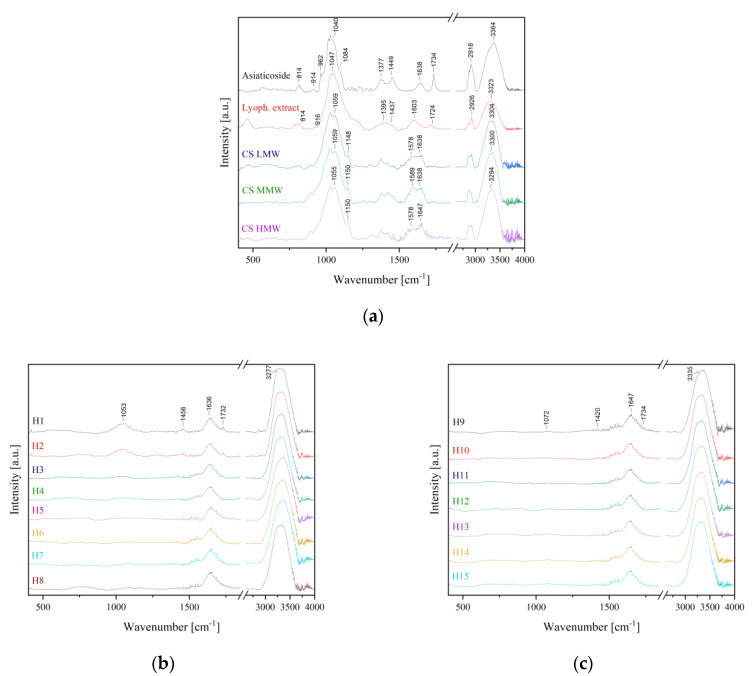
FTIR-ATR spectra of asiaticoside; lyophilized extract; LMW, MMW and HMW CS (**a**); hydrogels H1–H8 (**b**); and hydrogels H9–H15 (**c**).

In order to verify the successful grafting of the extract onto CS, the FTIR-ATR spectra of neat CSs, the extract (Figure 3a) and hydrogels H1–H15 were recorded (Figure 3b,c). The spectrum of CS can be easily used to identify the characteristic absorption bands: a broad band around 3300 cm^−1^ attributed to the overlapped peaks of the O-H and N-H stretching vibrations, the amide I (C=O stretching) and amide II (N-H bending) modes at 1638 and 1578 cm^−1^, respectively, and strong absorption bands at 1150 cm^−1^ and 1059 cm^−1^ due to the asymmetric stretching of the C-O-C bond and the C-O stretching vibration of secondary alcohols, respectively [22]. The spectrum of asiaticoside showed an absorption peak at 3364 cm^−1^ that can be assigned to O-H, a peak at 2918 cm^−1^ assigned to R-OH, peaks at 1734 and 1638 cm^−1^ assigned to C=O, and peaks at 962, 914 and 814 cm^−1^ assigned to -(CH_2_)_n_ bands [23]. In the spectrum of the extract, characteristic peaks assigned to bonds in the asiaticoside molecule, which confirmed its presence, were observed.

These characteristic peaks were also found in the hydrogel spectra. A low-intensity peak at 1732 cm^−1^, attributed to the C=O groups in asiaticoside, disappeared at lower concentrations in the spectra of hydrogels. Visible bands at 3277 cm, 1636 and 1053 cm^−1^ are the result of overlapping of the corresponding bands described above from the extract and CSs. The above indicates that the hydrogels were successfully synthesized.

**Figure 4:**
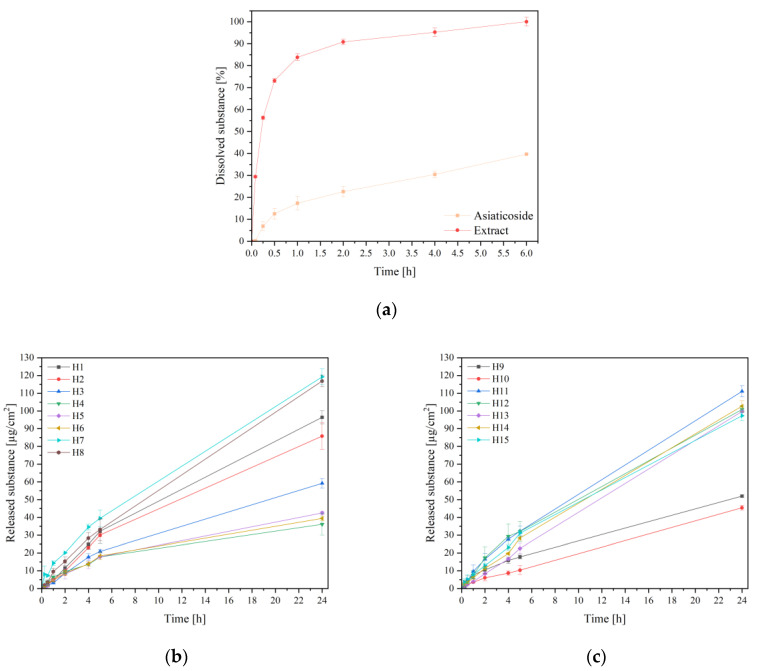

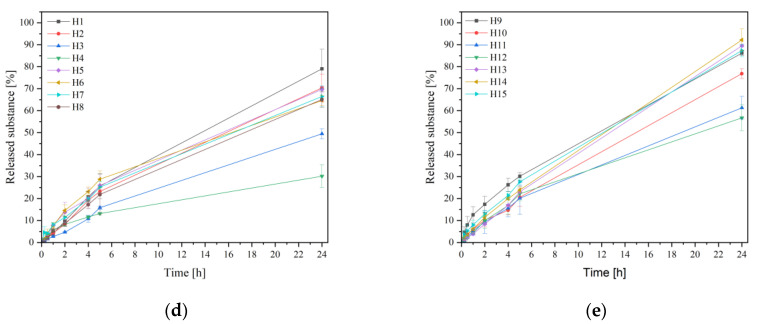
Release profiles of solid asiaticoside and from lyophilized extract (**a**); release profiles of asiaticoside (expressed in µg/cm^2^) from hydrogels H1–H8 (**b**) and H9–H16 (**c**); release profiles of asiaticoside (expressed in %) from hydrogels H1–H8 (**d**) and H9–H16 (**e**).

In vitro release studies were carried out (Figure 4) in order to estimate the asiaticoside release rate. It is known that asiaticoside is poorly water-soluble substance (Figure 4a) [24]. In the case of asiaticoside release from extracts, two distinct release stages were observed. In the first stage, in the first 1 h approximately, an accelerated and almost immediate release could be seen; this is known as the “burst effect”. This period was followed by a slower and more constant release stage that lasted up to 6 h and was recorded as a plateau (Figure 4a). The burst effect in the case of the extract results from the fact that it was subjected to lyophilization, which causes the amorphization of the ingredients, as well as the presence of other ingredients in the extract that increased asiaticoside dissolution.

In the case of hydrogel formulations H9, H13, H14 and H15, complete release of asiaticoside was observed within 24 h. Other formulations did not reach 80% release of the substance after 24 h (Figure 4d,e). In this case, the cumulative amount of released asiaticoside should also be taken into account. The greatest amount of asiaticoside was released from formulations H7, H8, and H11 (Figure 4b,c). Obviously, in this case, the statistically significant factors influencing the amount of asiaticoside released were the concentration of the extract and the concentration of CS (Figures S6 and S7, Supplementary Material). CS concentration is generally one of the most important factors influencing the amount or percentage of substance release. The more CS present, the more the viscosity of the hydrogel increases, slowing down the release (Figures S6 and S7, Supplementary Material). In this case, the MW of chitosan was not a statistically significant factor; however, a trend was observed that the release decreases with the increase in MW, which again is associated with an increase in viscosity with higher-MW chitosans.

As can be seen, the release profiles suggest that, for every formulation, the process might be satisfactorily correlated to and described by a zero-order kinetics model (Tables S3 and S4, Supplementary Material), which means that the drug release rate is independent of its concentration. The second best fit was with using the Korsmeyer–Peppas kinetic model, where *n* values were between 0.45 and 0.89, indicating that the release was controlled by coupled diffusion of the drug in the hydrated matrix and relaxation of the polymer. Such release kinetics from CS-based hydrogels are known and described in the literature [21,25]; therefore, CS can be successfully used as a carrier for the controlled release of active substances [26,27].

**Figure 5:**
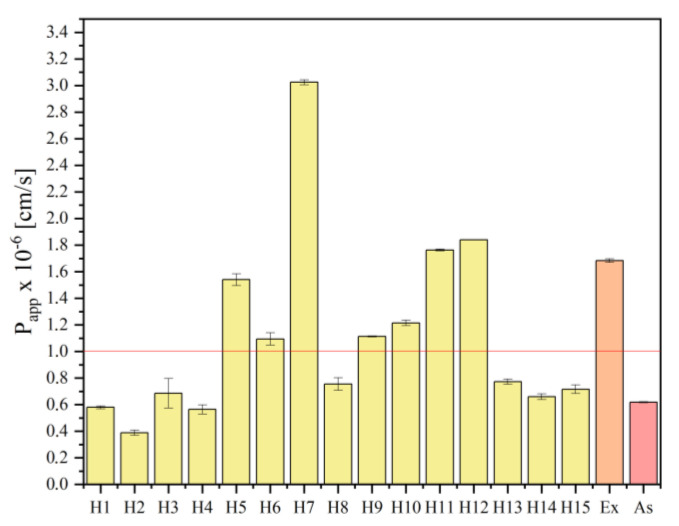
Apparent permeability values of asiaticoside from hydrogels H1–H15, from lyophilized extract and of standard sample, where ‘Ex’ stands for extract and ‘As’ for asiaticoside. The red line indicates P_app_ = 1 × 10^−6^ cm/s, i.e., substances with high permeability.

When the action of active compounds is intended to take place at the application site and does not require a systemic response, the skin provides an accessible and practical route for drug administration. However, it is important to keep in mind that biologically active substances must also reach the deeper layers of the skin in order to modify inflammatory signaling pathways. As a result, the presented research study included an assessment of the penetration of asiaticoside through biological membranes that simulate the skin barrier. It was possible to investigate the asiaticoside permeability by passive diffusion using the PAMPA skin model. Initially, it was shown that solvents do not affect the integrity of the membrane. The apparent permeability value (P_app_) for asiaticoside was 0.62 × 10^−6^ cm/s, which classifies it as a low-permeation component, and this has already been described in the literature with a suggestion that asiaticoside needs penetration promoters to penetrate deeper into the skin layers [28]. On the other hand, the value above 1 × 10^−6^ cm/s obtained regarding the penetration of asiaticoside from the extract suggests that the entire extract should be used and that it acts as an absorption promoter. The permeation coefficients for asiaticoside from all hydrogels are shown in Figure 5. When assessing the importance of the hydrogel components’ influence on the asiaticoside permeation, a trend can be observed that the membrane permeation decreases with increasing viscosity. On the other hand, an increase in the concentration of the extract only slightly increases the permeation of the substance, but both trends are not statistically significant (Figure S8, Supplementary Material).

**Figure 6:**
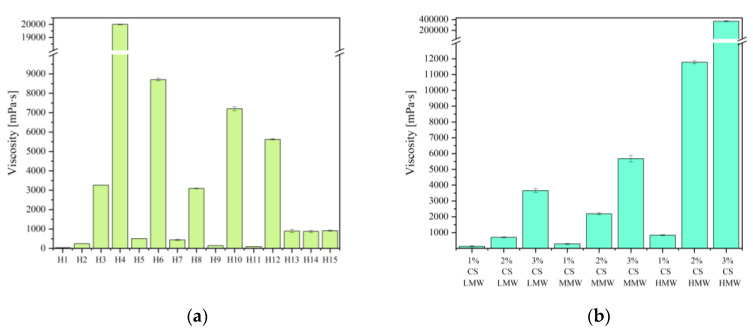
Viscosity of hydrogels H1–H15 (**a**) and chitosan bases (**b**).

The viscosity of systems is one of the most important determinants of bioadhesive properties [29]. Therefore, the viscosity of the systems was investigated (Figure 6). The MW and degree of deacetylation are the two basic characteristics of CS. The amount of free amino groups found in the CS macromolecule, which in turn dictates the polymer’s functionality, polarity, and water solubility, depends on the degree of deacetylation. On the other hand, the strength of the fiber or film and the viscosity of the solution are determined by the MW [30]. As the MW increases, the viscosity of the hydrogel increases (Figure 6b). Thus, the viscosity of the hydrogels is a result of the type of CS used. Obviously, with an increase in the concentration of CS and an increase in the MW, the viscosity of the hydrogel increased significantly, with a value of *p* of >0.05 (Figure S9, Supplementary Material). The tissue adhesion ability of chitosan hydrogels is mainly due to charge interactions, along with the formation of mutually entangled chains between two contacting interfaces [31]. Note that the adhesion of basic CSs may be relatively low compared to modified ones, which are formed by conjugating different adhesive agents to the CS backbone [32].

**Figure 7:**
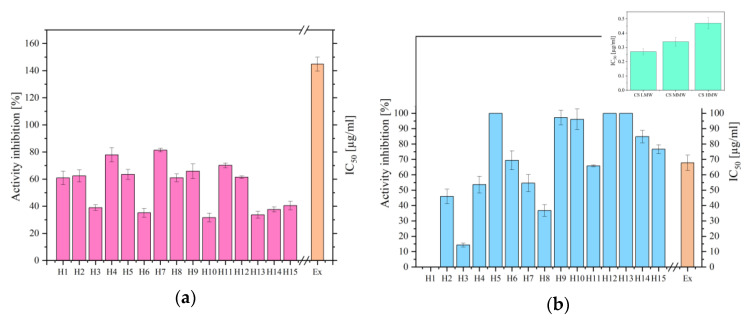
Antioxidant (**a**) and anti-hyaluronidase (**b**) activity of hydrogels H1–H15, where ‘Ex’ stands for extract.

Using in vitro models, the biological activities of the CS-based hydrogels were examined to see whether it was possible to scavenge DPPH radicals and inhibit hyaluronidase. These models were chosen to assess the anti-inflammatory effect of the system because hyaluronic acid plays a crucial role in the wound healing process, including hydration, anti-inflammation, and encouragement of cellular migration. All tested hydrogels showed antioxidant activity, but it was weaker than that of the extract itself, because CS is not a strong antioxidant. On the other hand, the strongest anti-inflammatory activities were observed for pure CS hydrogels, which were 100 times more potent than extract alone (Figure 7, Figures S10 and S11, Supplementary material). The process of reducing hyaluronidase activity could be associated with the interplay between amino groups (-NH_2_) of CS and carboxyl groups (-COOH) of the hyaluronic acid enzyme, as well as the increasing average size of the chitosan nanoparticles with an increasing molecular weight, perhaps resulting in modifications to the enzyme’s secondary structure [33,34]. Therefore, a significant increase in the inhibition of the breakdown of hyaluronic acid may indicate the combined action of active compounds from the extract of *C. asiatica*, specifically asiaticoside and chitosan molecules.

**Figure 8:**
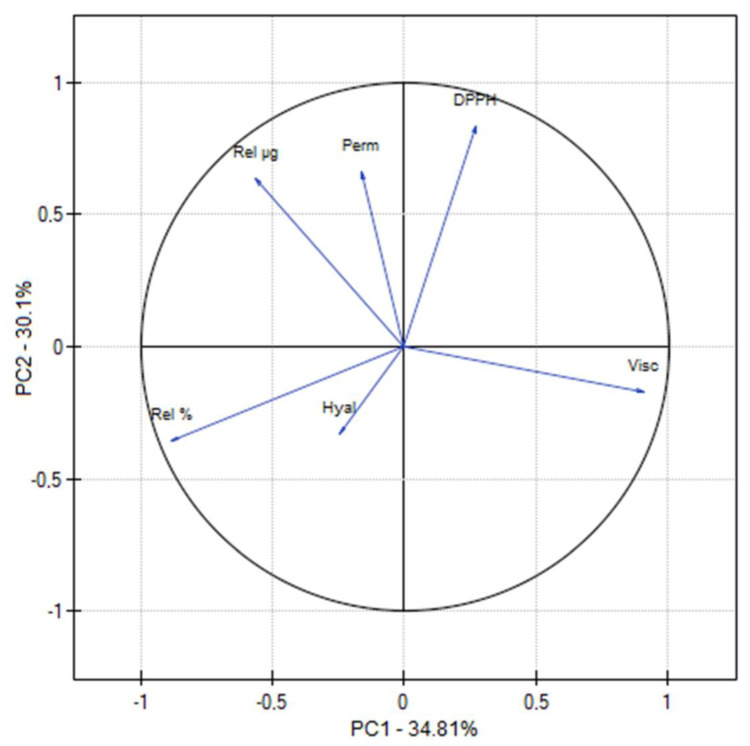
PCA for characteristics of hydrogels.

To evaluate the correlation of all experimental results, PCA analysis was performed (Figure 8; Table S5, Supplementary Material). A strong negative correlation was found between the viscosity of the hydrogels and the release of asiaticoside, which confirms the previously obtained results.

It was feasible to estimate the model and identify the ideal composition of the hydrogel on the basis of experimental research and statistical analysis. In order to obtain the best properties of hydrogels, 3% extract and 3% MMW chitosan should be used (Figure S12, Supplementary Material), which is the composition of the H12 hydrogel.

Additionally, the biological properties of the optimal hydrogel were assessed in the context of assessing microbiological activity and regenerative properties in a scratch test. In the case of microbiological activity, it is easy to see that the activity of the hydrogel depends largely on the content of the extract (Table 2 and Table 3). Importantly, the activity of the hydrogel against pathogenic bacteria living on the skin, including *Staphylococcus aureus*, was demonstrated (Table 3).

**Figure 9:**
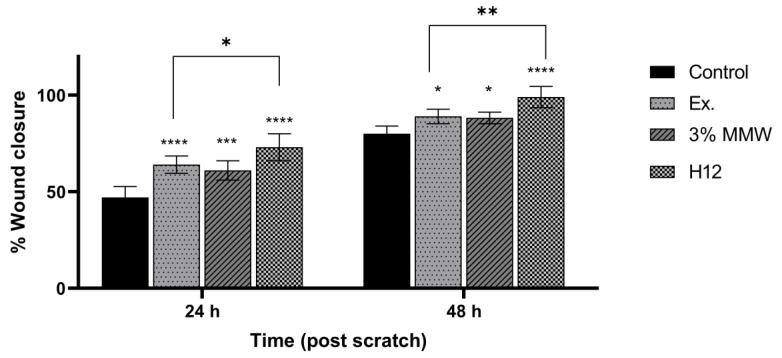
Time-course effect of *C. asiatica* lyophilized extract (Ex.), 3% MMW chitosan (3% CS MMW) and H12 hydrogel (H12) on post-scratch wound closure by human normal skin fibroblasts (Hs27 cells). Ex. and H12 contain equal concentrations of *C. asiatica* extract (details are given in Materials and Methods section). Results are expressed as means ± SD. Statistical significance was designated as (*) *p* < 0.05; (**) *p* < 0.01; (***) *p* < 0.001; (****) *p* < 0.001 (vs. control or vs. Ex. at the respective time points) using a two-way ANOVA followed by Tukey’s post hoc test.

A scratch test was performed in a cell line to observe the wound healing process, in which the cells polarize towards the wound, initiate protrusion, migrate and close the wound (Figure 9, Table 4). Both ingredients of H12 hydrogel, i.e., *C. asiatica* extract and 3% MMW chitosan, administered alone, stimulated migration of fibroblasts and wound-healing effects in a statistically significant manner. However, the H12 hydrogel had even stronger wound-healing properties and its percentage wound closure was as high as 73.4% and 99.0% after 24 and 48 h of incubation, respectively. Importantly, H12 hydrogel-induced closure of the wound area was statistically significantly more effective than wound-healing effect of the *C. asiatica* extract alone, which indicates the synergism between the ingredients of the investigated hydrogel.

Taking into account the results of experimental studies, it is worth noting the high clinical usefulness of the proposed formulation. In addition to the demonstrated biological effect on wound healing, it is worth noting the patient-friendly form of the hydrogel, which, thanks to its water base, is preferred by patients compared to fatty ointments. Nevertheless, in order to introduce the product into clinical use, it would be necessary to continue work on demonstrating its activity in vivo using an animal model and finally a human model.

## 3. Materials and Methods

### 3.1. Plant Material

Plant material, *Centellae asiaticae herba*, was purchased from NANGA (Blękwit, Poland).

### 3.2. Chemicals and Reagents

Asiaticoside (Phyproof^®^ Reference Substance), asiatic acid (Phyproof^®^ Reference Substance) and madecassic acid (Phyproof^®^ Reference Substance) were obtained from Sigma-Aldrich (Poznan, Poland). Excipients, such as chitosans (low molecular weight = LMW 20–300 cps, 1% in 1% acetic acid; medium molecular weight = MMW 200–800 cps; high molecular weight = HMW 800–2000 cps) wee supplied from Sigma-Aldrich (Poznan, Poland). Reagents for activity assays (2,2-Diphenyl-1-picrylhydrazyl (DPPH), sodium chloride, bovine serum, hexadecyltrimethylammonium bromide (CTAB), hyaluronic acid (HA)), dissolution studies (phosphate buffer) and bioadhesive tests (mucin from porcine stomach) were obtained from Sigma-Aldrich (Poznan, Poland). The hydration solution was obtained from Pion Inc. (Billerica, MA, USA), whereas HPLC grade acetonitrile and water were obtained from Merck (Darmstadt, Germany). High-quality pure water and ultra-high-quality pure water were prepared using a Direct-Q 3 UV Merck Millipore purification system.

### 3.3. Synthesis of Centellae asiaticae Extracts and Characteristion of Their Biological Activity

#### 3.3.1. Plant Extraction Using a Design of Experiment (DoE)

Using the Design of Experiments (DoE) approach, a factor experiment plan was developed for three independent variables, which were assigned three levels of values using a Box–Behnken plan (Statistica 13.3 software, TIBCO Software Inc., Palo Alto, CA, USA). As independent factors, the content of the extraction mixture, its temperature and the time of the extraction process were selected (Table 5).

An amount of 5.0 g of ground plant material was flooded with 100.0 mL of the appropriate extraction mixture, and using an ultrasonic bath (ultrasound power 120 W), the plant material was extracted 3 times for a certain period of time (Table 1). The extracts were combined and concentrated to 20.0 mL to give a drug/extract ratio (DER) of 1:4.

As the parameters used to assess extraction efficiency, the sum of content of active components (asiaticoside, asiatic acid and madecassic acid), the total content of phenolic compounds and antioxidants (DPPH scavenging assay) as well as the anti-inflammatory activities (inhibition of hyaluronidase activity) were selected.

#### 3.3.2. Determination of Selected Active Component Content and Total Phenolic Content (TPC)

The contents of main active compounds (asiaticoside, asiatic acid and madecassic acid) were determined by using the modified HPLC-Diode-Array Detection method. As equipment, an LC system (Dionex Thermoline Fisher Scientific, Waltham, MA, USA) with Chromeleon software version 7.0 was used. Separations were performed on a LiChrospher RP-18 column, with a 5 μm particle size, 250 mm × 4 mm (Merck, Germany). The detection was performed using a diode array detector at a maximum wavelength (λmax) of 200 nm. The following modifications to the pharmacopeial method were made: the mobile phase was composed of acetic acid 0.3% (A) and acetonitrile (B) with a gradient elution of 0–50 min 22–50% B, 50–55 min 80% B, 55–60 min 22% B. The flow rate of the mobile phase was 1.0 mL/min and the column temperature was maintained at 30 °C.

The total content of phenolic components was determined by using a method described previously [35].

#### 3.3.3. Determination of Biological Activity

##### Antioxidant Activity

The antioxidant activity was determined by using an assay with 2,2-Diphenyl-1-picrylhydrazyl (DPPH). The procedure has been described previously [35].

##### Anti-Hyaluronidase Activity

The procedure of hyaluronidase inhibition was determined by a turbidimetric method described previously [35].

##### Microbiological Activity

Research was carried out in accordance with the methodology described previously [36]. *C. asiaticae* extracts were dissolved 1% DMSO in order to obtain a final concentration of 100 μg/mL. The remaining “hydrogel” samples were applied directly to the well.

### 3.4. Preparation of Hydrogels Containing Centellae asiaticae Extracts

#### 3.4.1. Hydrogel Preparation Using a Design of Experiment (DoE)

The composition of the hydrogels (amount of extract, amount of chitosan and molecular weight of chitosan) was selected on the basis of Design of Experiment (DoE) data and a Box–Behnken plan (Statistica 13.3 software, TIBCO Software Inc., Palo Alto, CA, USA) and is presented in Table 6.

To prepare the hydrogels, water was weighed into a beaker and the appropriate amount of extract was added and stirred at 500 rpm for 5 min. Chitosan was then added and dissolved by adding 1% acetic acid. The hydrogel was stirred for 24 h before testing.

The parameters used to assess the efficiency of hydrogel production were release of active compounds, penetration of active compounds, bioadhesive properties, antioxidant activity (determined by the DPPH method), and anti-inflammatory activity (expressed by the degree of inhibition of the hyaluronidase enzyme).

#### 3.4.2. Release of Active Compounds

In vitro release experiments were performed on the hydrogels with the use of vertical Franz diffusion cells (PermeGear, Inc., Hellertown, PA, USA), each containing 5 mL of acceptor solution (phosphate buffer; pH = 5.5). The cells were equipped with regenerated cellulose membranes (Nalo Cellulose^®^, Kalle GmbH, Wiesbaden, Germany) with a pore diameter of ca. 25 Å. The membranes were immersed in the acceptor fluid at 37.0 ± 0.5 °C for 24 h before the experiment. Gel samples (1.0 mL) were placed in the donor compartment and spread evenly on the surface of the artificial membrane. The effective diffusion area of the employed cells was 0.64 cm^2^. The receptor fluid during the test was stirred at 400 rpm, and its temperature was set at 37.0 ± 0.5 °C. Samples (2.0 mL) were taken from the acceptor compartment at appropriate time points and immediately replaced with an equal volume of fresh acceptor fluid. The asiaticoside concentrations in the collected samples were determined with the HPLC-DAD method described above.

#### 3.4.3. Penetration of Active Compounds

The permeability of standards, extract and hydrogels was investigated by using the Skin PAMPA method (skin parallel artificial membrane permeability assay). The Skin PAMPA model consists of a two-chamber PAMPA sandwich composed of two 96-well plates. The top plate contains the lipid-impregnated skin-mimetic membrane. Before use, pre-coated Skin PAMPA™ sandwich plates (Pion Inc.) were hydrated overnight by placing 200 μL of the hydration solution in each well (Hydration Solution, Pion Inc.). Each experiment was repeated at least three times using six replicates on each plate. The amount of permeated active compounds after 3 h was determined using the HPLC method described above.

The apparent permeability coefficient (*P_app_*) was calculated via the following equation:

$$P_{app}=\frac{-ln\left(1-\frac{C_{A}}{C_{equilibrium}}\right)}{S\times \left(\frac{1}{V_{D}}+\frac{1}{V_{A}}\right)\times t}$$

where *V_D_*—donor volume, *V_A_*—acceptor volume, *C_equilibrium_*—equilibrium concentration $C_{equilibrium}=\frac{C_{D}\times V_{D}+C_{A}\times V_{A}}{V_{D}+V_{A}}$, *C_D_*—donor concentration, *C_A_*—acceptor concentration, *S*—membrane area, *t*—incubation time (in seconds).

Moreover, we looked into whether the integrity of the biomimetic artificial membrane may be compromised by solvents. Each solvent was added to a well, and the wells were left to incubate for a minimum of 6 h—longer than the tests that used the model solutions. After the solvents were drawn out of the wells, cotton paper was used to gently remove any remaining residue from the membrane’s surface. Next, using piroxicam as the model permeant—for which prior exact data are available—a routine skin permeability assay was carried out.

#### 3.4.4. Bioadhesive Properties

A viscometric method was used to predict bioadhesive properties [27]. The viscosity of the prepared hydrogels was measured using an AMETEK Brookfield DV2T viscometer (Hadamar-Steinbach, Germany).

#### 3.4.5. Determination of Biological Activity

Antioxidant and anti-hyaluronidase activities were assessed according to the methodology described in Section 3.3.3.

A microbiological assay was performed according to the description in Section 3.3.3. Hydrogel samples were applied directly to the well.

##### Wound Healing Assay

Human skin fibroblasts Hs27 (CRL-1634) were purchased from the American Type Culture Collection (Manassas, VA, USA) and maintained in DMEM-high glucose supplemented with 10% FBS, penicillin (100 U mL^−1^) and streptomycin (100 µg mL^−1^). On the day of the experiment, cells were collected from monolayers using trypsin/EDTA. The wound closure ability of Hs27 cells was examined using a scratch assay. Hs27 cells were seeded onto a 6-well plate at a concentration of 1 × 10^5^ cells mL^−1^ and the cells were cultivated in DMEM-high glucose supplemented with 10% FBS, penicillin (100 U mL^−1^) and streptomycin (100 µg mL^−1^). When the confluence of cells reached about 90%, a vertical linear scratch was created in the monolayer with a sterile pipette tip. Cells were washed three times with phosphate-buffered saline (PBS) to remove all cellular debris, and the fresh medium containing 2% FBS (control group) or fresh medium containing *C. asiatica* lyophilized extract (Ex.), 3% MMW chitosan solution (3% CS MMW) or hydrogel H12 (H12) was added to respective wells. Working solutions of 3% CS MMW and H12 were obtained by mixing 3% CS MMW chitosan solution or H12 hydrogel with cell culture medium in a ratio of 2:98 (*v*/*v*). A working solution of the *C. asiatica* lyophilized extract was obtained by direct dissolution of the extract in a culture medium in such a way as to obtain the same concentration of the extract in the H12 solution. Afterwards, images of the scratch were taken after 0 h, 24 h, and 48 h using an Olympus CKX53 microscope coupled with an XM10 digital camera (Olympus). Before taking the images, the medium was aspirated (and saved) and the cells were washed with PBS. The experiments were performed at least in duplicate. The scratch area at the beginning of the experiment (0 h) was considered 100%. The open wound area was measured with NIH ImageJ software (Bethesda, Rockville, MD, USA) (https://imagej.net/nih-image/, access date: 1 September 2023). The percentage of wound closure was calculated using the following formula:

$$Wound\;closure\left(\%\right)=\frac{open\;wound\;area\;at\;0\;h-open\;wound\;area\;at\;24\;h\;or\;48\;h}{open\;wound\;area\;at\;0\;h}\times 100\%$$

## 4. Conclusions

The obtained hydrogel containing asiaticoside-rich *Centella asiatica* extract based on chitosan meets the functional criteria in terms of rheological properties, including appropriate mucoadhesion at the site of application, penetration of the active compound through the skin barrier based on passive diffusion and biological activity expressed by microbiological, anti-inflammatory and regenerating effects. In order to introduce the product into clinical use, it would be necessary to continue work on demonstrating the effect in vivo in an animal and, ultimately, a human model.

## Figures and Tables

**Table 1:** Phytochemical and biological characteristics of extracts E1–E15.

No.	Active Components Content (mg/1 g Plant Material)				TPC	Antioxidant Activity	Anti-Inflammatory Activity
	Asiaticoside (AS)	Asiatic Acid (AA)	Madecassic Acid (MA)	Sum	(mg GAE/1 g Plant Material)	IC_50_ (µg/mL)	IC_50_ (mg/mL)
E1	10.48 ± 0.09	0.04 ± 0.01	0.02 ± 0.01	10.54 ± 0.10	14.56 ± 0.52	481.48 ± 22.89	137.19 ± 4.11
E2	9.54 ± 0.42	5.96 ± 0.01	1.44 ± 0.01	16.93 ± 0.42	12.16 ± 0.53	774.11 ± 14.10	194.95 ± 6.02
E3	8.59 ± 0.03	0.40 ± 0.01	0.11 ± 0.01	9.09 ± 0.03	18.66 ± 0.60	307.05 ± 19.53	70.39 ± 2.18
E4	10.78 ± 0.01	0.07 ± 0.01	0.02 ± 0.01	10.87 ± 0.01	15.97 ± 0.46	534.53 ± 23.06	91.53 ± 3.84
E5	8.98 ± 0.02	0.04 ± 0.01	0.01 ± 0.01	9.03 ± 0.02	13.87 ± 0.62	584.64 ± 21.21	103.40 ± 4.17
E6	9.74 ± 0.17	6.15 ± 0.01	1.54 ± 0.01	17.44 ± 0.18	14.81 ± 0.72	893.77 ± 34.00	127.39 ± 4.92
E7	9.68 ± 0.01	0.06 ± 0.01	0.01 ± 0.01	9.75 ± 0.02	17.21 ± 0.67	513.86 ± 22.87	91.60 ± 3.89
E8	10.85 ± 0.01	0.43 ± 0.01	0.11 ± 0.01	11.39 ± 0.01	15.90 ± 0.52	742.75 ± 36.33	98.54 ± 4.61
E9	9.63 ± 0.03	0.83 ± 0.01	0.23 ± 0.01	10.69 ± 0.01	14.26 ± 0.53	378.79 ± 16.00	91.37 ± 2.61
E10	11.35 ± 0.09	5.10 ± 0.01	1.35 ± 0.01	17.80 ± 0.09	16.88 ± 0.64	482.94 ± 7.69	88.12 ± 0.35
E11	11.47 ± 0.01	4.47 ± 0.01	1.12 ± 0.01	17.05 ± 0.02	15.61 ± 0.68	372.37 ± 4.29	87.15 ± 2.57
E12	11.51 ± 0.09	5.18 ± 0.01	1.36 ± 0.01	18.05 ± 0.09	16.45 ± 0.56	383.05 ± 16.97	75.46 ± 3.53
E13	12.12 ± 0.05	3.09 ± 0.01	0.90 ± 0.01	16.11 ± 0.02	17.64 ± 0.61	373.61 ± 8.24	82.16 ± 2.09
E14	12.24 ± 0.06	3.19 ± 0.01	0.93 ± 0.01	16.36 ± 0.03	16.88 ± 0.73	343.77 ± 9.32	90.89 ± 2.63
E15	12.32 ± 0.01	3.21 ± 0.01	0.95 ± 0.01	16.48 ± 0.01	17.60 ± 0.59	358.69 ± 10.55	65.38 ± 2.49

**Table 2:** Microbiological activity of the optimized *C. asiatica* extract.

	Growth Inhibition Zone (mm)
*Enterococcus faecalis* ATTC 29212	11.0 ± 2.0
*Staphylococcus aureus* ATCC 25923	10.0 ± 2.0
*Staphylococcus pyrogenes* ATCC 19615	12.0 ± 2.0
*Escherichia coli* ATCC 25922	11.0 ± 2.0
*Pseudomonas aeruginosa* ATCC 15442	8.0 ± 1.0
*Streptococcus mutans* ATCC 25175	11.0 ± 2.0
*Staphylococcus epidermidis* ATCC 25175	13.0 ± 2.0
*Enterobacter aerogenes* ATCC 13048	12.0 ± 1.0

**Table 3:** Microbiological activity of the chitosan base and hydrogel H12.

	3% CS	Hydrogel H12
	Growth Inhibition Zone (mm)	
*Enterococcus faecalis* ATTC 29212	5.0 ± 1.0	1.0 ±1.0
*Staphylococcus aureus* ATCC 25923	5.0 ± 1.0	1.0 ± 1.0
*Staphylococcus pyrogenes* ATCC 19615	6.0 ± 1.0	2.0 ± 1.0
*Escherichia coli* ATCC 25922	5.0 ± 1.0	1.0 ± 1.0
*Pseudomonas aeruginosa* ATCC 15442	6.0 ± 1.0	1.0 ± 1.0
*Streptococcus mutans* ATCC 25175	5.0 ± 1.0	1.0 ± 1.0
*Staphylococcus epidermidis* ATCC 25175	7.0 ± 1.0	2.0 ± 1.0
*Enterobacter aerogenes* ATCC 13048	6.0 ± 1.0	1.0 ± 1.0

**Table 4:** Representative images of wound-healing properties of *C. asiatica* lyophilized extract (Ex.), 3% MMW chitosan (3% CS MMW) and H12 hydrogel (H12).

	Control	Ex.	3% CS MMW	H12
0 h	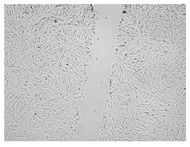	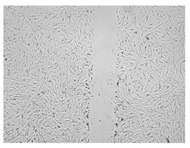	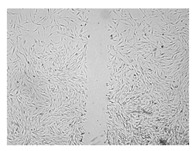	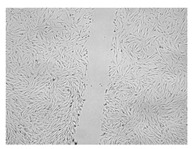
24 h	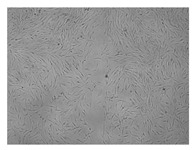	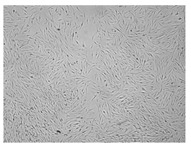	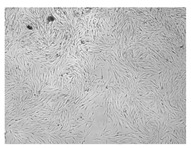	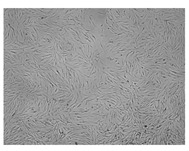
48 h	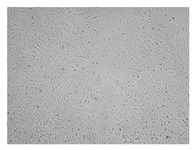	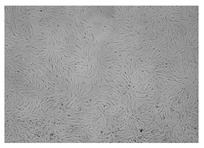	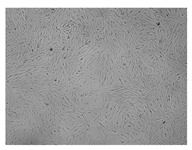	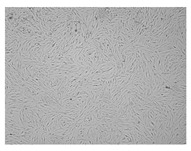

**Table 5:** Factorial extraction process experiment plan.

No.	% Methanol in the Extraction Mixture	Temperature	Time (min)
E1	50	30	60
E2	90	30	60
E3	50	70	60
E4	90	70	60
E5	50	50	30
E6	90	50	30
E7	50	50	90
E8	90	50	90
E9	70	30	30
E10	70	70	30
E11	70	30	90
E12	70	70	90
E13	70	50	60
E14	70	50	60
E15	70	50	60

**Table 6:** Factorial hydrogel preparation experiment plan.

No.	Chitosan MW	Chitosan Concentration	Extract Concentration
H1	100	1	2
H2	1100	1	2
H3	100	3	2
H4	1100	3	2
H5	100	2	1
H6	1100	2	1
H7	100	2	3
H8	1100	2	3
H9	600	1	1
H10	600	3	1
H11	600	1	3
H12	600	3	3
H13	600	2	2
H14	600	2	2
H15	600	2	2

## Data Availability

Data is contained within the article and Supplementary Material.

## Supplementary Materials

The following supporting information can be downloaded at: https://www.mdpi.com/article/10.3390/ijms242417229/s1.
