# Supplementary material for: Chitosan-Based Hydrogels for Controlled Delivery of Asiaticoside-Rich Centella asiatica Extracts with Wound Healing Potential
Source: Int J Mol Sci. 2023 Dec 7;24(24):17229. doi: 10.3390/ijms242417229 (PMC10743457; doi:10.3390/ijms242417229)
Supplement: Supplementary file 1 [file ijms-24-17229-s001.zip › ijms-2748675-supplementary.pdf]

Chitosan-based hydrogels for controlled delivery of asiaticoside-rich *Centella asiatica* extract

Supplementary material

Table S1. Validation parameters of HPLC method

Parameter	**Asiaticoside**	**Madecassic acid**	**Asiatic acid**
Linearity: y = ax + b			
$a \pm S_a$	0.0492 ± 0.0003	0.6260 ± 0.0127	0.0992 ± 0.0020
$b \pm S_b$	insignificant (α=0.05)	insignificant (α=0.05)	insignificant (α=0.05)
Correlation coefficient (r)	0.9999	0.9994	0.9994
Range of linearity [μg/mL]	79.2–792.0	5.2–52.0	32.8–328.0
Intra-day precision, RSD (<5% required) = repeatability			
The lowest	1.18	2.74	2.74
The middle	0.38	0.34	0.34
The lowest	1.06	0.09	0.09
Limit of detection (LOD) [μg/mL]	12.12	2.43	15.36
Limit of quantification (LOQ) [μg/mL]	36.72	7.38	46.54

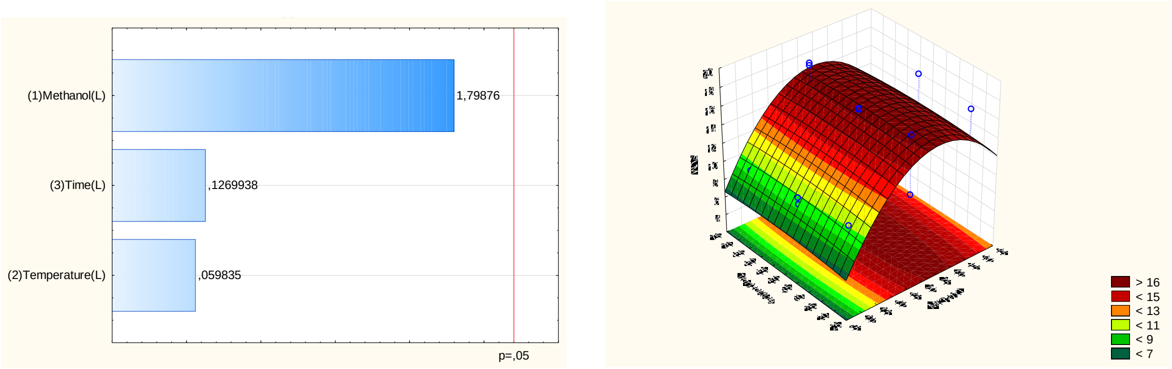

(a) (b)

Figure S1. Statistical analysis for sum of active components content in extracts E1-E15: (a) Pareto plot of standardized effects for sum of active components content in extracts E1-E15; (b) Response surface plots presenting the dependence of methanol content in the extraction mixture and extraction temperature on the sum of active components content in extracts for constant time at level 60 minutes.

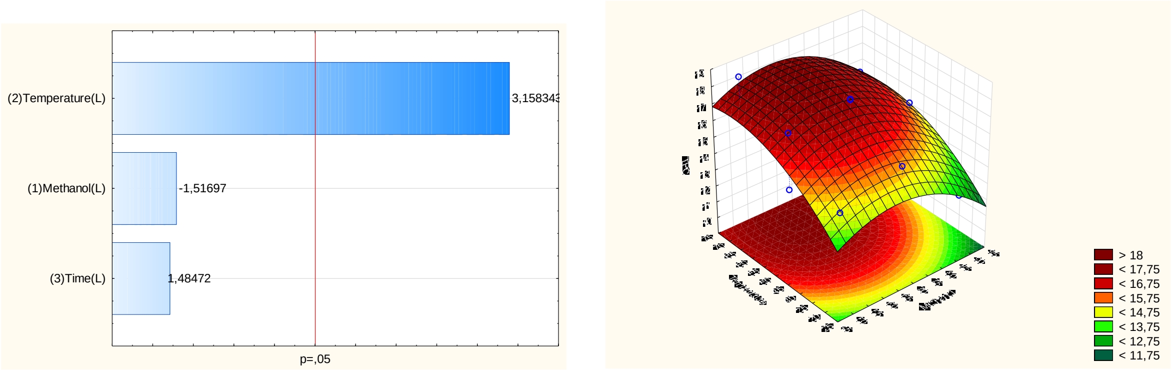

(a) (b)

Figure S2. Statistical analysis for total phenolic content in extracts E1-E15: (a) Pareto plot of standardized effects for total phenolic content in extracts E1-E15; (b) Response surface plots presenting the dependence of methanol content in the extraction mixture and extraction temperature on the total phenolic content in extracts for constant time at level 60 minutes.

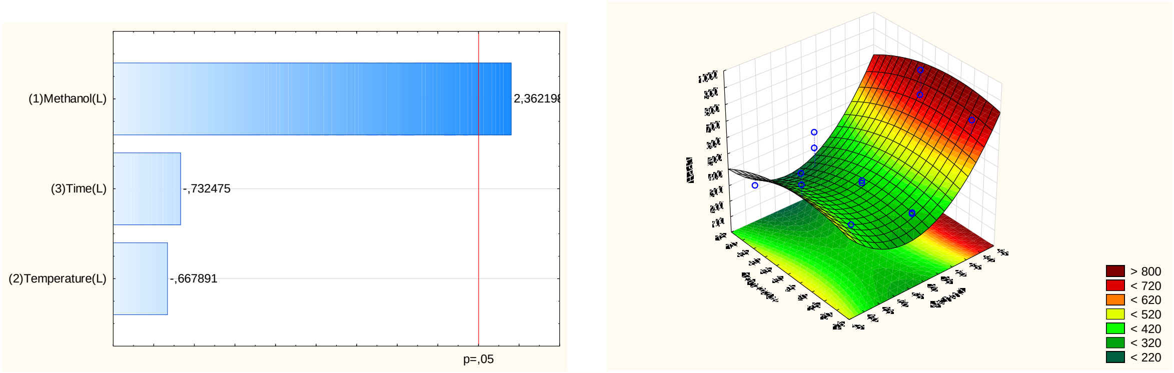

(a) (b)

Figure S3. Statistical analysis for antioxidant activity of extracts E1-E15 measured by DPPH method: (a) Pareto plot of standardized effects for antioxidant activity; (b) Response surface plots presenting the dependence of methanol content in the extraction mixture and extraction temperature on antioxidant activity of extracts for constant time at level 60 minutes.

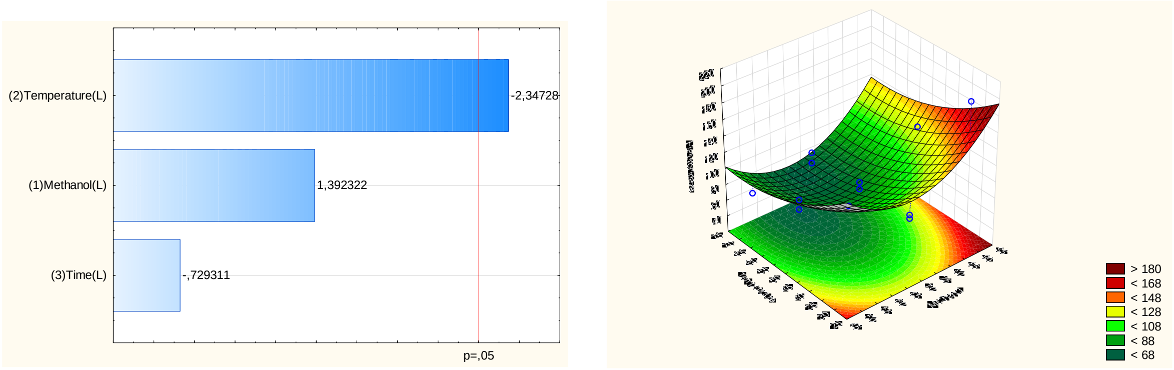

(a) (b)

Figure S4. Statistical analysis for anti-inflammatory activity of extracts E1-E15: (a) Pareto plot of standardized effects for anti-inflammatory activity of extracts E1-E15; (b) Response surface plots presenting the dependence of methanol content in the extraction mixture and extraction temperature on anti-inflammatory activity of extracts for constant time at level 60 minutes.

Table S2. Correlation matrix

	AS, AA, MA content	TPC	DPPH	Hyal
**AS, AA, MA content**	1,0000	0,0158	0,0484	0,0833
**TPC**	0,0158	1,0000	-0,6027	-0,8209
**DPPH**	0,0484	-0,6027	1,0000	0,6907
**Hyal**	0,0833	-0,8209	0,6907	1,0000

statistically significant relationships are marked in red

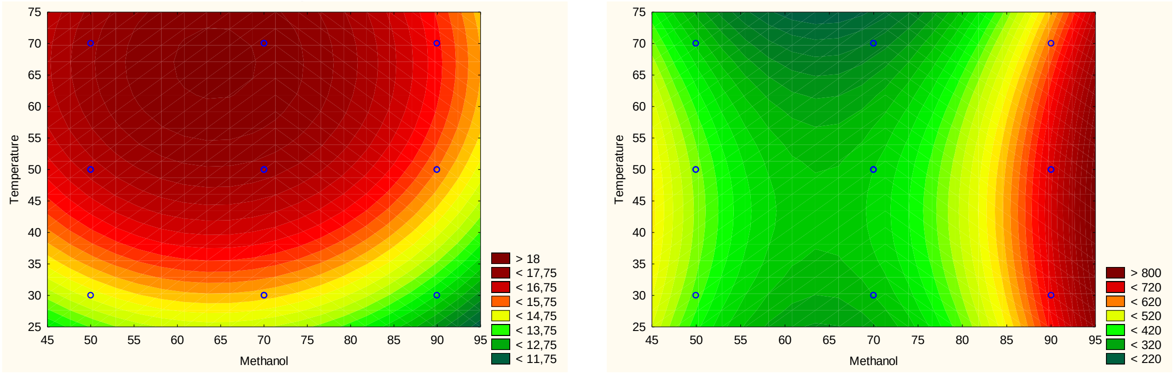

(a) (b)

Figure S5. Prediction of the optimization model for obtaining extracts based on effect with positive sign like sum of active components content and TPC (a) and those with negative sing like DPPH and hyaluronidase assays (b).

Table S3. Parameters of mathematical models fitted to the release profiles (expressed in μg/cm²) of formulations H1-H15

Formulation	Mathematical model							
No.	Zero-order kinetic		First-order kinetic		Higuchi kinetic		Korsmeyer-Peppas kinetic	
	K	R^2	K	R^2	K	R^2	R^2	n
H1	**3.98**	**0.98**	0.16	0.60	18.97	0.62	**0.97**	**0.60**
H2	**3.53**	**0.98**	0.15	0.61	8.05	0.63	**0.97**	**0.58**
H3	**2.43**	**0,97**	0.15	0.59	5.66	0.65	**0.93**	**0.55**
H4	**1.39**	**0.90**	0.11	0.51	3.81	0.80	**0.97**	**0.48**
H5	**1.71**	**0.94**	0.13	0.55	4.28	0.71	**0.93**	**0.52**
H6	**1.57**	**0.92**	0.12	0.54	4.08	0.76	**0.96**	**0.50**
H7	**4.72**	**0.98**	0.13	0.51	11.02	0.65	**0.93**	**0.57**
H8	**4.48**	**0.99**	0.15	0.58	10.53	0.60	**0.98**	**0.61**
H9	**2.03**	**0.97**	0.11	0.57	4.88	0.68	**0.97**	**0.48**
H10	**1.85**	**1.00**	0.14	0.68	3.87	0.55	**0.91**	**0.47**
H11	**4.53**	**0.99**	0.16	0.53	10.12	0.62	**0.94**	**0.63**
H12	**4.10**	**0.98**	0.15	0.56	9.47	0.65	**0.98**	**0.60**
H13	**4.15**	**1.00**	0.15	0.56	8.39	0.52	**0.95**	**0.57**
H14	**4.23**	**1.00**	0.15	0.65	8.98	0.56	**0.98**	**0.57**
H15	**3.95**	**0.99**	0.14	0.60	8.82	0.61	**0.97**	**0.55**

the most fitting mathematical model is shown in bold

Table S4. Comparison of asiaticoside release profiles (expressed in %) from H1-H15 hydrogels using factors f_1 and f_2

	H1	H2	H3	H4	H5	H6	H7	H8	H9	H10	H11	H12	H13	H14
H1														
H2	f_1=10.43<br>f_2=65.66													
H3	f_1=39.81<br>f_2=34.85	f_1=33.54<br>f_2=40.70												
H4	f_1=52.14<br>f_2=30.39	f_1=46.35<br>f_2=35.03	f_1=35.95<br>f_2=66.42											
H5	f_1=13.35<br>f_2=97.92	f_1=10.50<br>f_2=67.76	f_1=64.63<br>f_2=35.35	f_1=94.80<br>f_2=30.79										
H6	f_1=21.81<br>f_2=93.70	f_1=21.05<br>f_2=62.43	f_1=68.76<br>f_2=34.01	f_1=98.82<br>f_2=29.79	f_1=9.85<br>f_2=89.75									
H7	f_1=17.88<br>f_2=91.61	f_1=14.12<br>f_2=70.94	f_1=62.59<br>f_2=36.04	f_1=91.54<br>f_2=31.36	f_1=8.27<br>f_2=96.46	f_1=10.66<br>f_2=83.64								
H8	f_1=16.74<br>f_2=56.24	f_1=8.41<br>f_2=77.19	f_1=41.64<br>f_2=44.86	f_1=69.86<br>f_2=38.16	f_1=13.97<br>f_2=57.59	f_1=16.75<br>f_2=54.07	f_1=12.89<br>f_2=59.57							
H9	f_1=29.92<br>f_2=41.03	f_1=43.46<br>f_2=35.09	f_1=95.85<br>f_2=22.69	f_1=95.29<br>f_2=20.03	f_1=31.11<br>f_2=40.38	f_1=27.90<br>f_2=42.20	f_1=32.76<br>f_2=39.53	f_1=52.40<br>f_2=32.35						
H10	f_1=12.34<br>f_2=73.10	f_1=14.60<br>f_2=87.64	f_1=55.26<br>f_2=38.74	f_1=82.91<br>f_2=33.50	f_1=17.43<br>f_2=76.04	f_1=24.85<br>f_2=68.75	f_1=19.38<br>f_2=80.65	f_1=16.02<br>f_2=68.56	f_1=28.07<br>f_2=36.75					
H11	f_1=21.80<br>f_2=50.10	f_1=14.67<br>f_2=64.02	f_1=33.98<br>f_2=49.54	f_1=61.25<br>f_2=41.51	f_1=18.91<br>f_2=51.12	f_1=20.89<br>f_2=48.44	f_1=17.89<br>f_2=52.57	f_1=7.13<br>f_2=78.72	f_1=38.15<br>f_2=30.10	f_1=15.46<br>f_2=58.77				
H12	f_1=21.39<br>f_2=49.03	f_1=15.05<br>f_2=62.06	f_1=31.84<br>f_2=50.63	f_1=61.36<br>f_2=42.26	f_1=19.92<br>f_2=50.00	f_1=21.88<br>f_2=47.45	f_1=18.91<br>f_2=51.38	f_1=9.49<br>f_2=75.18	f_1=38.92<br>f_2=39.66	f_1=20.77<br>f_2=57.21	f_1=9.10<br>f_2=97.55			
H13	f_1=13.21<br>f_2=91.52	f_1=17.35<br>f_2=61.34	f_1=69.73<br>f_2=33.70	f_1=96.01<br>f_2=29.45	f_1=25.14<br>f_2=86.74	f_1=33.80<br>f_2=99.10	f_1=28.67<br>f_2=81.11	f_1=23.40<br>f_2=53.32	f_1=24.93<br>f_2=42.65	f_1=17.16<br>f_2=67.33	f_1=31.27<br>f_2=47.85	f_1=32.07<br>f_2=46.89		
H14	f_1=15.56<br>f_2=60.87	f_1=23.52<br>f_2=48.36	f_1=85.85<br>f_2=29.20	f_1=98.95<br>f_2=25.68	f_1=21.51<br>f_2=59.30	f_1=28.66<br>f_2=63.87	f_1=23.18<br>f_2=57.35	f_1=31.22<br>f_2=43.56	f_1=20.37<br>f_2=51.91	f_1=20.10<br>f_2=51.49	f_1=39.21<br>f_2=39.91	f_1=40.96<br>f_2=39.23	f_1=9.50<br>f_2=65.09	
H15	f_1=16.66<br>f_2=53.63	f_1=28.82<br>f_2=43.98	f_1=93.83<br>f_2=27.27	f_1=98.35<br>f_2=24.03	f_1=18.81<br>f_2=52.49	f_1=21.08<br>f_2=55.76	f_1=20.72<br>f_2=51.04	f_1=36.85<br>f_2=39.97	f_1=11.44<br>f_2=58.51	f_1=24.84<br>f_2=46.51	f_1=45.19<br>f_2=36.84	f_1=47.02<br>f_2=36.25	f_1=17.16<br>f_2=56.60	f_1=10.37<br>f_2=79.09

similar profiles marked in red

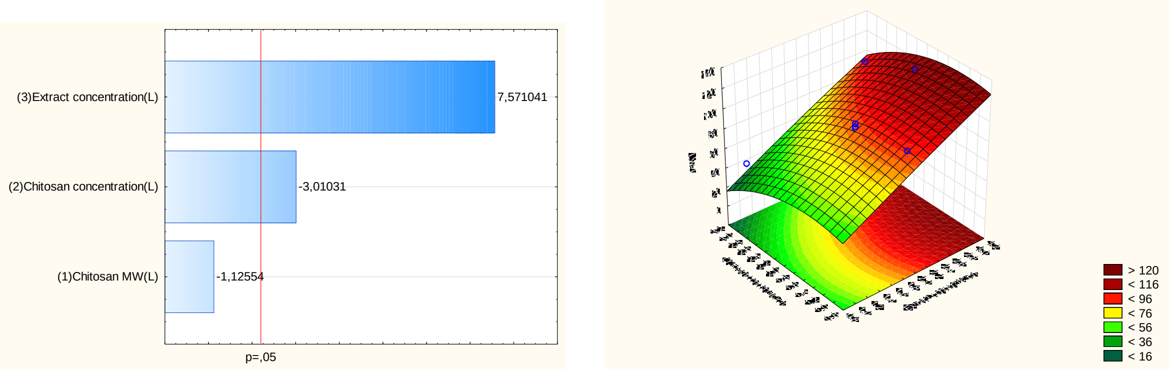

(a) (b)

Figure S6. Statistical analysis for release studies: (a) Pareto plot of standardized effects for asiaticoside release (in µg/cm²); (b) Response surface plots presenting the dependence of extract concentration and chitosan concentration on the asiaticoside release from hydrogels (in µg/cm²) for constant chitosan molecular weight at level 600.

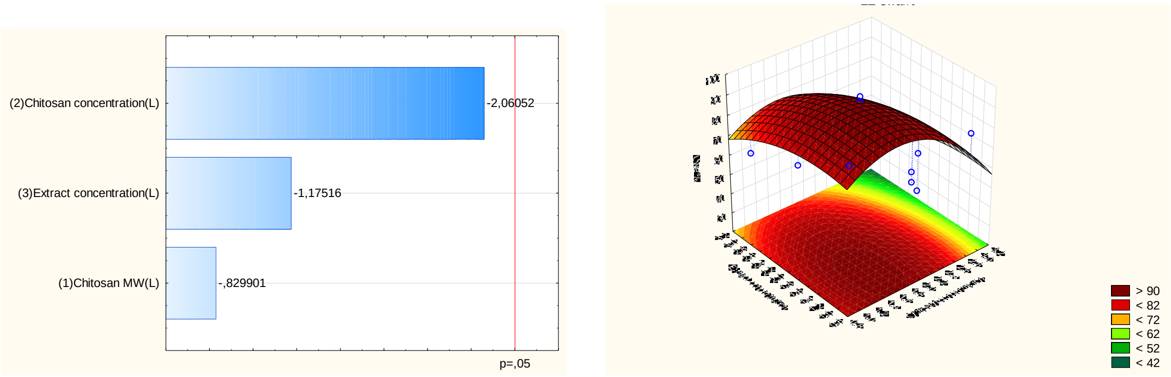

(a) (b)

Figure S7. Statistical analysis for release studies: (a) Pareto plot of standardized effects for asiaticoside release (in %); (b) Response surface plots presenting the dependence of chitosan concentration and extract concentration on the asiaticoside release from hydrogels (in %) for constant chitosan molecular weight at level 600.

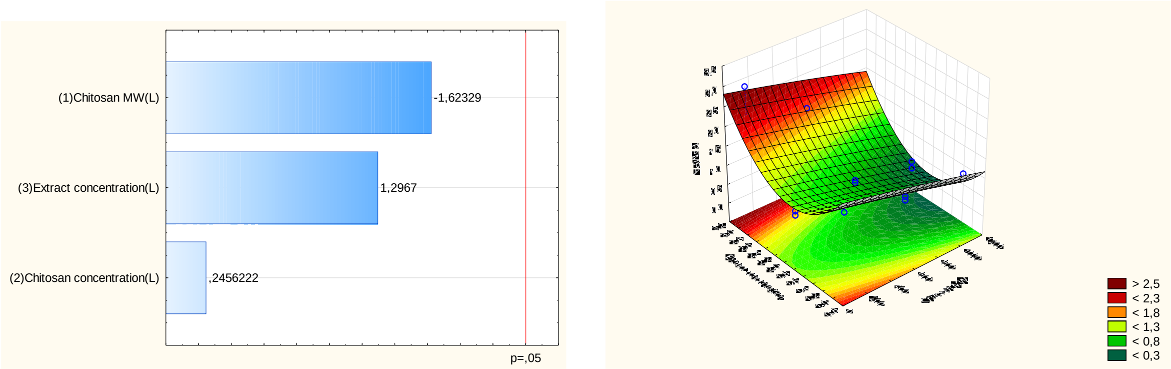

(a) (b)

Figure S8. Statistical analysis for asiaticoside permeability: (a) Pareto plot of standardized effects for asiaticoside permability; (b) Response surface plots presenting the dependence of chitosan molecular weight and extract concentration on the asiaticoside permeability for constant chitosan concentration at level 2%.

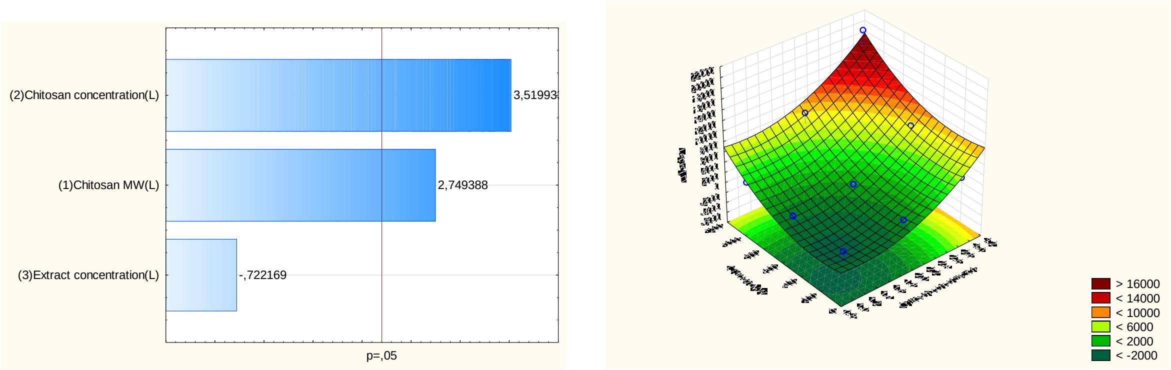

(a) (b)

Figure S9. Statistical analysis for hydrogels' viscosity: (a) Pareto plot of standardized effects for mucoadhesive properties of hydrogels' viscosity; (b) Response surface plots presenting the dependence of chitosan concentration and chitosan molecular weight on the hydrogels' viscosity for constant extract concentration at level 2%.

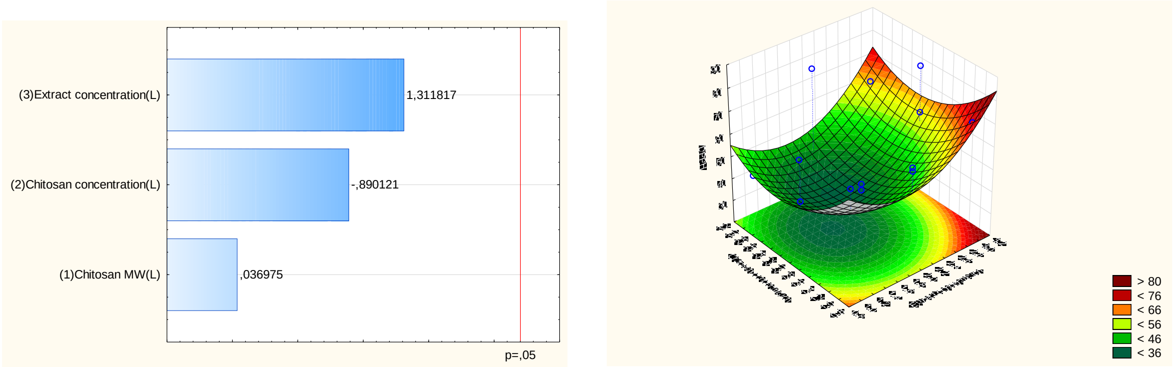

(a) (b)

Figure S10. Statistical analysis for antioxidant activity of hydrogels: (a) Pareto plot of standardized effects for antioxidant activity of hydrogels H1-H15; (b) Response surface plots presenting the extract and chitosan concentration on antioxidant activity for chitosan' medium molecular weight.

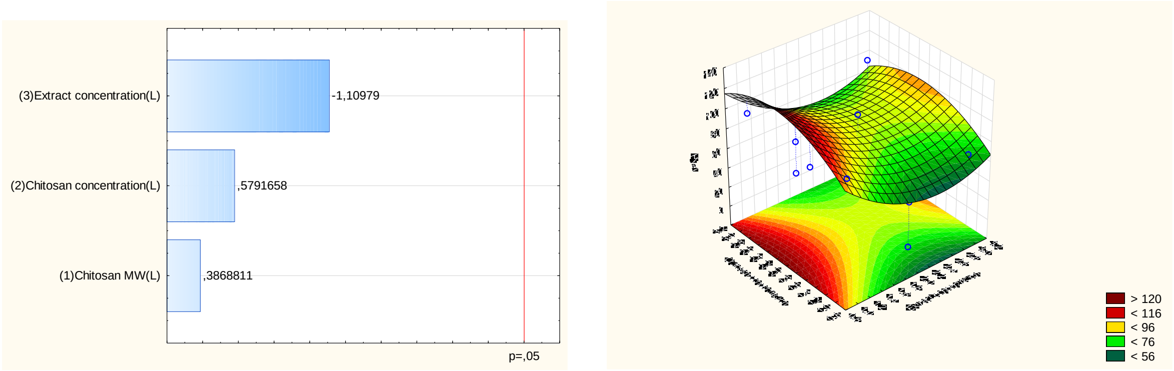

(a) (b)

Figure S11. Statistical analysis for anti-inflammatory activity of hydrogels: (a) Pareto plot of standardized effects for anti-inflammatory activity of hydrogels H1-H15; (b) Response surface plots presenting the extract and chitosan concentration on antioxidant activity for chitosan' medium molecular weight.

Table S5. Correlation matrix

	**Asiaticoside release [$\mu g/cm^2$]**	**Asiaticoside release [%]**	**Asiaticoside permeability**	**Hydrogel viscosity**	**DPPH**	**Hyal**
**Asiaticoside release [$\mu g/cm^2$]**	1.0000	0.2267	0.3014	-0.5462	0.2640	-0.2444
**Asiaticoside release [%]**	0.2267	1.0000	-0.1103	-0.7135	-0.4420	0.3330
**Asiaticoside release [$\mu g/cm^2$]**	0.3014	-0.1103	1.0000	-0.1740	0.4368	0.2731
**Hydrogel viscosity**	-0.5462	-0.7135	-0.1740	1.0000	0.0819	-0.0088
**DPPH**	0.2640	-0.4420	0.4368	0.0819	1.0000	-0.2125
**Hyal**	-0.2444	0.3330	0.2731	-0.0088	-0.2125	1.0000

statistically significant relationships are marked in red

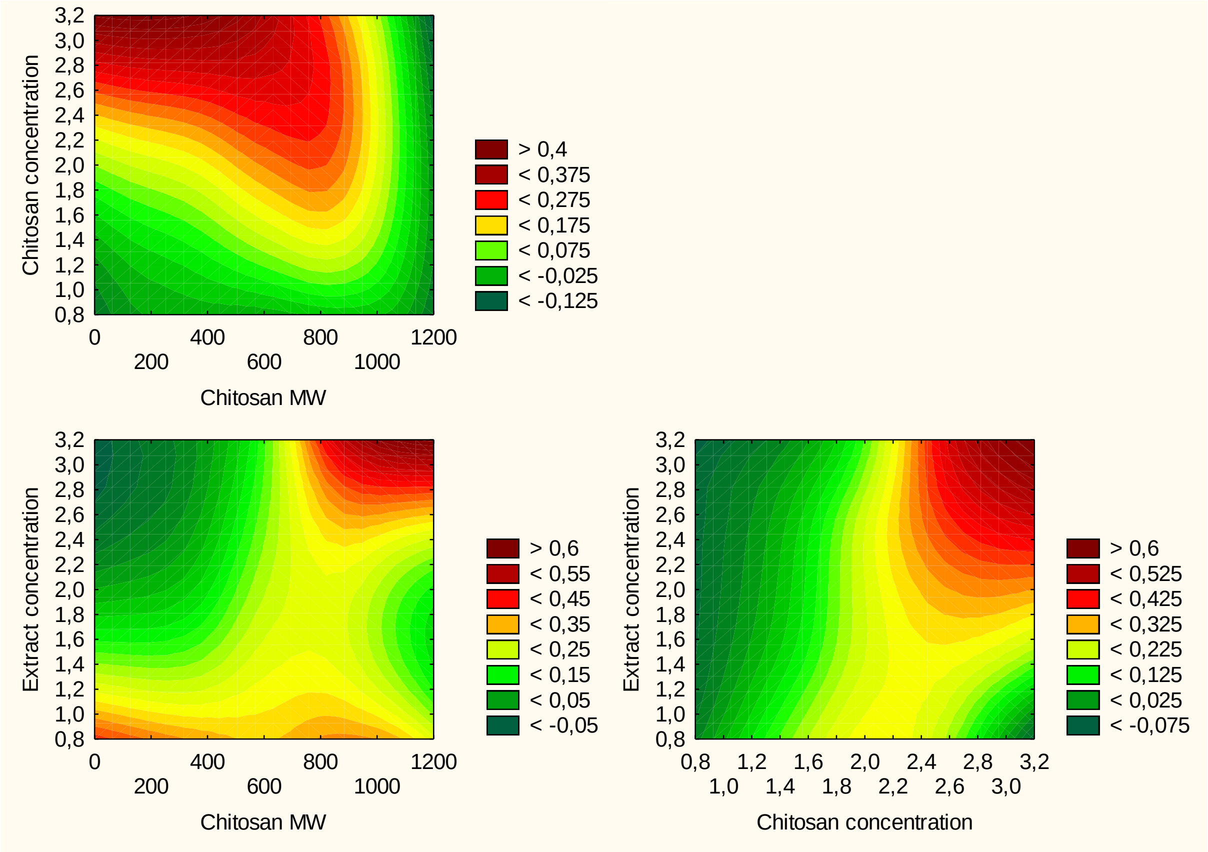

Figure S12. Prediction of the optimization model for obtaining hydrogels.
